# Analysis of potential factors affecting allografts contamination at retrieval

**DOI:** 10.1007/s10561-017-9667-9

**Published:** 2017-10-17

**Authors:** A. Paolin, C. Romualdi, L. Romagnoli, D. Trojan

**Affiliations:** 1 Fondazione Banca dei Tessuti di Treviso onlus, Treviso, Italy; 2 Department of Biology, University of Padova, Padova, Italy; 3 Areta International, Varese, Italy

**Keywords:** Tissue banking, Microbiology, Retrieval, Allografts, Risk factors

## Abstract

The microbiological contamination of retrieved tissues has become a very important topic and it is a critical aspect in the safety of allografts, especially from multi-tissue donors whose tissues are frequently contaminated as a consequence of retrieval. We analysed a total of 10,107 tissues, 8178 musculoskeletal and 1929 cardiovascular tissues, retrieved from 978 multi-tissue donors. Of these, 159 heart-beating donors (HBD) were also organ donors, while the remaining 819 non-heart-beating donors (NHBD) were tissue donors only. A multivariate logistic model was used to determine the factors affecting contamination risk during retrieval. In the model, the dependent variable was the presence/absence of contamination while the covariates included were: gender, type of donor, age of donor, cause of death, previous skin donation, cadaver time, number of people attending the retrieval, number of tissues retrieved. Moreover, a second log-linear model was used to determine the number of strains isolated per tissue. Tissue contamination was statistically correlated with gender, type of donor, cadaver time, number of people attending the retrieval and season. In conclusion, to minimize the risk of bacterial contamination, aseptic techniques should be used at retrieval, with the number of retrieval team members kept to a minimum. In addition, cadaver time should be as short as possible and the donor should be refrigerated within a few hours after death.

## Introduction

Microbiological contamination is a key topic and understanding its leading causes is still a challenging task. There are many standards and guidelines to improve safety during tissue retrieval and processing. Vicentino et al. (2009) have demonstrated that contamination originates from the donor, the environment and the operator and that patients develop infections that are related to the implanted tissues. In the literature, there are many articles describing the mechanisms underlying the microbiological contamination of human tissues, but the multitude of possible variables involved makes it difficult to determine the triggering factors and the extent of their contribution. The risk of bacterial contamination is always present during the harvesting process. However, knowing the causes and applying adequate procedures can curb bacterial growth. Vehmeyer et al. (2002) have analysed the factors that influence the contamination rate of allografts and blood samples, showing that the risk of graft contamination increases with each extra team member in the case of bone donors. In addition, Lannau et al. (2015) have analysed several variables in a multiple regression model in order to determine the factors that influence the contamination risk during retrieval. Terzaghi et al. (2015) have reported a significant effect on contamination incidence according to the number of staff members involved in the retrieval. The aim of our study was to analyse several variables potentially involved in the bacteriological contamination of 10,107 tissues retrieved from 978 consecutive cadaveric donors, in order to clearly define which factors play a role in bacterial growth and to what extent.

## Materials and methods

### Data collection

10,107 consecutive tissues, 8178 musculoskeletal (MST) and 1929 cardiovascular tissues (CVT), retrieved from 978 consecutive multi-tissue donors were analysed. Of these, 159 heart-beating donors (HBD) were also organ donors, while the remaining 819 non-heart-beating donors (NHBD) were tissue donors only. All retrievals were based on requirements of the Italian Guidelines, approved by the National Transplant Centre. Our retrieval team of physicians and technicians harvested the tissues in the operating theatre after organ retrieval in HBD, and within 24 h of cardiac arrest in NHBD. Prior to tissue retrieval the skin underwent surgical scrubbing with chlorhexidine solution and shaving followed by an additional application of chlorhexidine and povidone iodine. Skin was the first tissue retrieved, followed by CVT and MST. Donors were selected and screened following strict criteria, in accordance with European directives and Italian guidelines. After retrieval all tissues were rinsed with isotonic saline solution that was then sampled (8 ± 10 ml) and submitted to microbiological cultures without filtering. All procedures were carried out at room temperature.

### Microbiological analysis

Microbiological cultures for aerobic and anaerobic bacteria, fungi/yeasts were carried out using BD BACTEC™/Alert Fluorescent Test Technology plus aerobic/F Medium and anaerobic/F culture vials, Soybean-Casein Digest Broth (BD, Becton, Dickinson and Company, New Jersey). Culture bottles were incubated at 36.5 °C for 7 days. Each vial contained a chemical sensor to detect increased CO_2_ produced by the growth of microorganisms and fluorescence, which was subsequently monitored by a BACTEC/Alert fluorescent series instrument. Culture bottles showing evidence of growth after 7 days were gram stained, sub-cultured on blood agar plates and incubated for 48 h at 35/38 °C in a normal atmosphere, a 5% CO_2_-enriched atmosphere, and an anaerobic atmosphere. The microorganisms in the test sample inoculated in the BACTEC vial metabolize the substrates producing CO_2_. Increased fluorescence caused by higher amounts of CO_2_ is detected by the BACTEC fluorescent series instrument. The analysis of the rate and amount of CO_2_ increase enables the BACTEC fluorescent series instrument to determine if the vial is positive, i.e., if the test sample contains viable microorganisms. Samples were then processed under a biohazard class-II laminar flow and all bacteria were identified with the standard biochemical procedure. Finally, an antibiogram was drawn up for each bacterium isolated and the Minimal Inhibitory Concentration estimated in the standard media used in clinical practice.

Samples were also cultured in a Lowenstein–Jensen medium to isolate mycobacteria. Microbiological cultures and analyses were carried out by an accredited in-hospital microbiology laboratory and interpreted by a microbiologist with specific expertise.

### Statistical analysis

A two-step approach was used to identify the factors affecting tissue contamination. In the first step, a multivariate logistic regression model with mixed effects was used in order to identify the factors affecting contamination risk during retrieval. In the model, the dependent variable was the presence/absence of contamination while the covariates included were: gender, type of donor, age of donor, cause of death, previous skin donation, cadaver time, number of people attending the retrieval, number of tissues retrieved and the season during which the tissues were retrieved. Moreover, given the presence of multiple tissues harvested from the same donor, we included a random effect determining the repeated measures. In the second step, we used a log-linear multivariate model in order to explain the number of contaminations at retrieval per tissue. Automatic selection via stepwise regression was used to select the best set of variables in the model. Statistical analyses were performed with R statistic software.

## Results

All variables analysed are summarized in Table 1.

**Table 1:** All variables analysed

Donor type	Gender	No.	%	Total
NHBD	Male	690	70.5	819
	Female	129	13.2	
HBD	Male	83	8.5	159
	Female	76	7.8	

	Mean	Min	Max	SD
Donor age (years)				
Male NHBD	42.6	0	65	14.12
Female NHBD	39.8	0	65	15.76
Male HBD	43.2	16	65	15.49
Female HBD	46.6	18	65	11.51
Cadaver time (h)	15.16	1	24	5.93
No. of people attending the retrieval	3.79	2	6	0.84
No. of tissues retrieved per donor	14.54	1	29	6.39

Cause of death	No.	%
Trauma	514	52.57
Heart attack	335	34.25
Stroke	112	11.45
Asphyxia	12	1.23
Drowning	5	0.51

Skin retrieval prior to MST and CVT retrieval	Yes	No
Season	Summer (from May to September)	Winter (from October to April)

5801 of 10,107 tissues retrieved were contaminated (57.4%), specifically, out of 1929 CVT 1461 (75.7%) were contaminated while out of 8178 MST 4340 (53.1%) proved positive after retrieval.

**Fig. 1:**
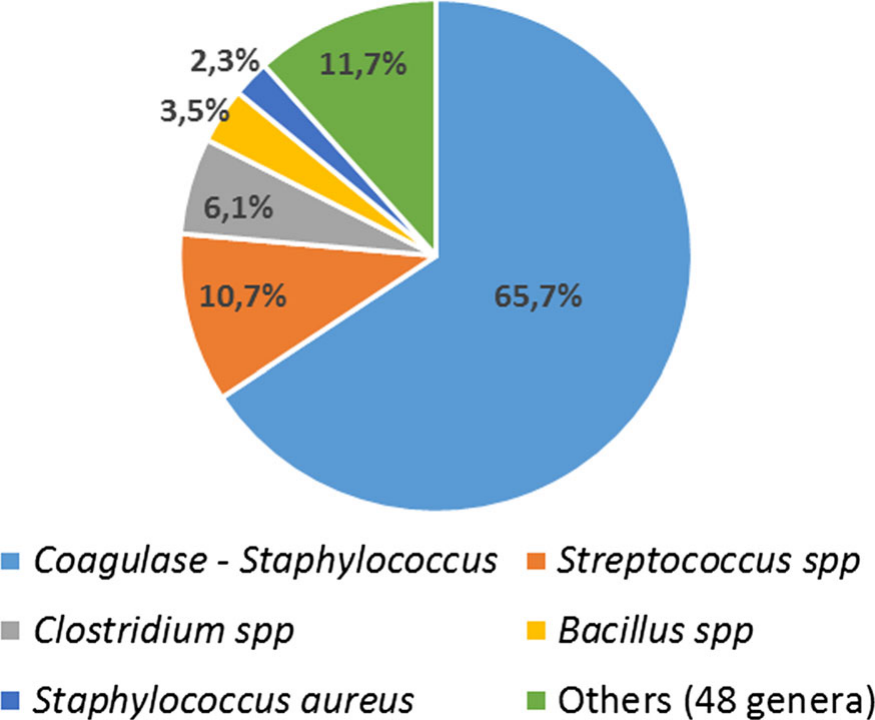
Percentages of genera detected upon retrieval

Of 5801 tissues 4773 were contaminated by one single strain (82.3% of total tissues contaminated), 796 by 2 strains (13.7% of total tissues contaminated), and 232 tissues by more than 2 strains (4% of total tissues contaminated). 7084 strains were isolated (1.22 strains per tissue on average). As already reported in previous papers (Deijkers et al. 1997; Ireland and Spelman 2005; Jashari et al. 2007; Paolin et al. 2017; Tabaku et al. 2004; Vehmeyer et al. 2002), the main strains isolated in allografts were *coagulase negative Staphylococci* (65.7%), *Streptococcus* spp. (10.7%), *Clostridium* spp. (6.1%), *Bacillus* spp. (3.5%) and *Staphylococcus aureus* (2.3%) (Fig. 1).

### Factors affecting tissue contamination

The multivariate logistic regression model identified four factors that are statistically correlated with tissue contamination upon retrieval. These are: gender, donor type, cadaver time and number of people attending retrieval. The risk of contamination is 1.58 times higher in male donors compared to female, 2.40 times higher in NHBD compared to HBD, 1.03 times higher for each extra hour of cadaver time and 1.28 times higher for each additional member of the retrieval team (Table 2).

**Table 2:** Logistic regression model

Variables	OR	Lower CI	Higher CI	*p* value
Gender				
Male	1.58	1.17	2.13	3.09e−03
Female	–	–	–	–
Donor type				
NHB	2.40	1.61	3.55	1.45e−05
HB	–	–	–	–
Cadaver time (h)	1.03	1.01	1.05	1.56e−03
Number of people attending the retrieval	1.28	1.07	1.53	6.66e−03

The table shows Odds Ratio (OR), Lower and Higher Confidence Interval (CI) and *p* value for each variable analysed. CI = 95%

The log-linear model has allowed us to identify four variables that statistically correlate with tissue contamination at retrieval. These are: gender, cadaver time, number of people attending the retrieval and time of year. Specifically, the risk of a new contamination is increased by a factor of 1.24 in male donors compared to female donors, 1.03 for each extra hour of cadaver time, 1.15 for each person attending the retrieval, and is decreased by 0.83 times in winter compared to summer (Table 3).

**Table 3:** Log-linear model

Variables	OR	Lower CI	Higher CI	*p* value
Gender				
Male	1.24	1.10	1.39	3.51e−04
Female	–	–	–	–
Cadaver time (h)	1.03	1.02	1.03	1.40e−11
No. of people attending the retrieval	1.14	1.07	1.23	4.21e−05
Season				
Winter	0.83	0.76	0.90	2.98e−05
Summer	–	–	–	–

The table shows Odds Ratio (OR), Lower and Higher Confidence Interval (CI) and *p* value for each variable analysed. CI = 95%

**Fig. 2:**
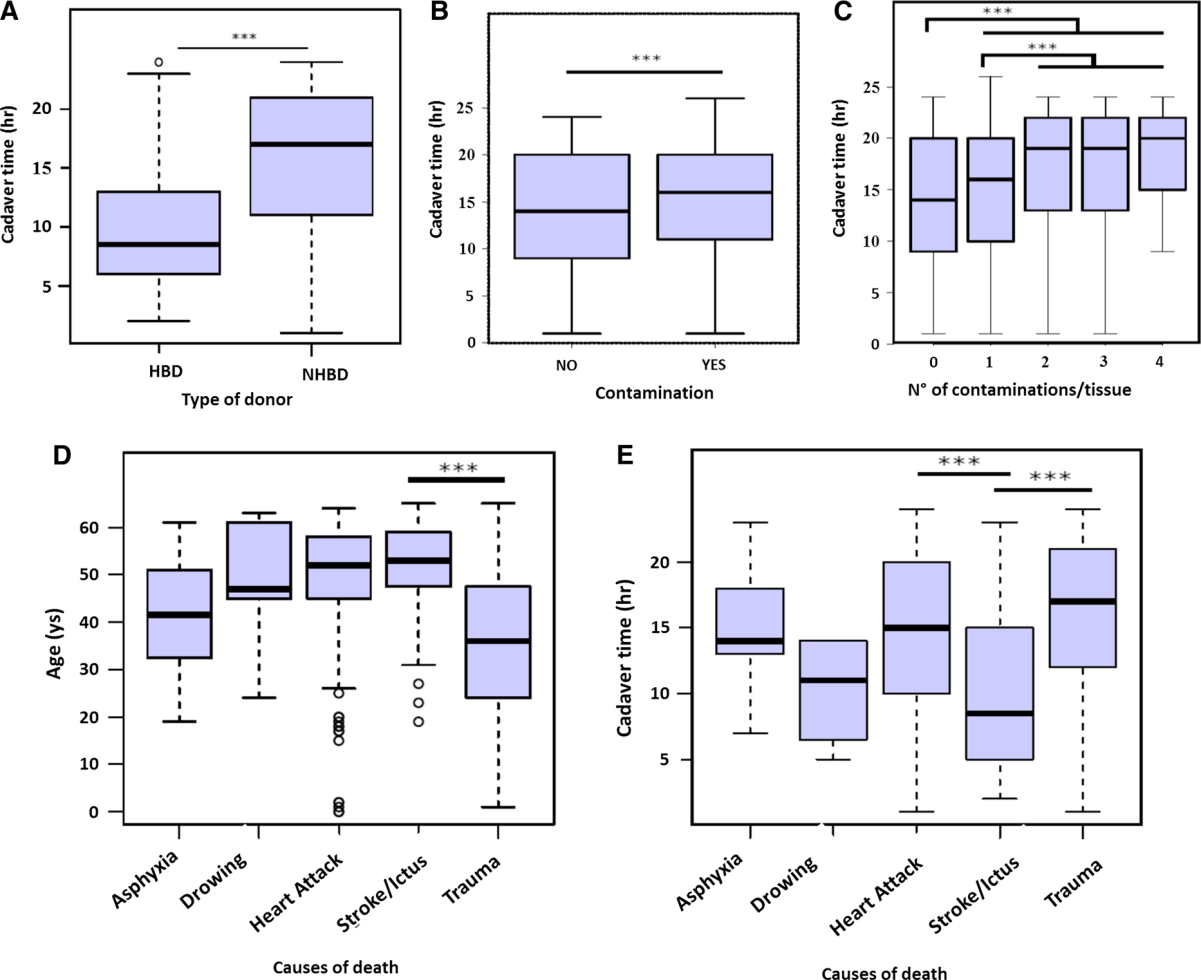
**a**–**e** Box plots of the most significant associations among variables. The line inside the box represents the median. The bar with asterisks means that there is a significant difference in the average between the classes (*p* value < 0.001)

Figure 2 shows the most significant associations (*p* value < 0.001) among the variables included in the analysis.

Cadaver time was significantly longer in NHBD compared to HBD (Fig. 2a) and significantly associated with the presence of contamination as well as the number of contaminants per tissue (Fig. 2b, c), i.e., the longer the cadaver time the higher the risk of contamination and the probability of multiple contaminations per tissue.

Trauma donors were significantly younger compared to donors whose cause of death was stroke (Fig. 2d). Cadaver time was significantly longer in donors whose cause of death was either trauma or heart attack in comparison to donors with stroke (Fig. 2e). The remaining causes of death (asphyxia and drowning) did not show any correlation with the other variables due to the paucity of data available.

## Discussion

In our sample the multivariate logistic model identified four variables which show a statistical correlation with the presence of contamination. These are: gender, donor type, cadaver time and the number of people attending the retrieval. The remaining variables included in the statistical analysis did not show any significant correlation. The first two variables (gender and donor type) are strictly correlated one with the other since the donors in our sample were mainly male NHBD (70.5% of total donors) whose cause of death was trauma in 52.63% cases. Trauma increases the risk of contamination in our sample by 2.4 times as reported by Deijkers et al. (1997) who found that the risk of graft contamination with highly pathogenic microorganisms increases 3.4 times after death due to trauma. Similarly, Martinez et al. (1985) claim that seeding of the blood-stream could take place in victims of extensive trauma or in patients submitted to invasive emergency procedures, such as our donors who are victims of road accidents and heart attacks. Conversely, Forsell and Liesman (2000) believe that the lack of correlation between the cause of death and contamination in their sample of 1036 donors from seven tissue banks in the USA is due either to the classification system used, unsuitable for the identification of the effect of cause of death on positive microbiological cultures, or to the infinite number of possible causes of death that does not allow for a correct classification based on macro-categories.

Cadaver time is the third variable with significant correlation, showing a contamination risk increasing by 1.03 times for every extra hour.

Cadaver time is significantly longer in NHBD (Fig. 2a) than HBD. This means that there is a greater exposure of the cadaver to the environment and to external factors with the consequent higher risk of contamination. NHBD in our sample died predominantly outside of hospitals and were removed from place of death, be it a street or a home, hours later, after a prolonged warm ischemic time which promotes the growth and migration of bacteria into the blood compartment in the period prior to the refrigeration of the bodies, after transportation to the referring hospital mortuary. HBD tissues were retrieved immediately after the removal of organs, only a few hours after circulatory arrest, whereas NHBD tissues were retrieved on average 17 h after blood circulation had stopped. Van Kats et al. (2010) also confirmed a significant relationship between warm ischemic time and contamination at retrieval. Finally, the lower number of tissues harvested from donors who had died of stroke compared to the high number of tissues from donors who had died of trauma or heart attack is due to the fact that most of the former have also donated organs as well. The number of people attending retrieval is the last significant variable affecting the contamination of tissues highlighted in our first model of analysis. Specifically, each extra person in the retrieval team accounts for an increased risk of contamination by 1.28 times. In the operating theatre there is the possibility of operator-dependent contamination; microorganisms originating from normal skin flora could be related to contamination during the harvesting procedure. Our results are comparable to those of other studies: Forsell and Liesman (2000) have demonstrated that the number of people attending retrieval increases the bioburden, and that the statistical analysis of recovery cultures can be a powerful tool that may be used to identify possible problems within any bank’s recovery procedures or techniques. Harvesting all the tissues that donors are potentially suitable to provide is time consuming and requires a team of at least 4/5 people, thereby increasing the risk of tissue contamination at retrieval as evidenced by Lannau et al. (2015) in a sample of 281 cadaveric donors. Retrieval teams consisting of only three members have a lower incidence of positive cultures than teams of fewer or more members (Forsell and Liesman 2000). Vehmeyer et al. (2002) showed that the risk of graft contamination increases with each extra team member (OR 1.9), similarly Terzaghi et al. (2015) found a significant correlation between contamination and the number of staff members retrieving MST as shown by Deijkers et al. (1997) and Segur et al. (2000) proving that the number of operators in the retrieval team is a determinant factor in the bacterial contamination rate of bone allografts.

The second model of analysis has highlighted a correlation between the number of strains isolated in each tissue and the selected variables. Results show that gender, cadaver time and number of people attending retrieval significantly affect not only the possible presence of microorganisms but also the number of isolated microorganisms in each single tissue. Specifically, a more protracted cadaver time implies a higher risk of contamination and a higher probability that the tissue is contaminated by multiple strains. Accordingly, NHBD show a higher contamination rate and a higher number of tissues with multiple contaminations, as already previously reported (Paolin et al. 2017). Moreover, this model of analysis has also shown that the period of the year during which retrieval takes place also plays a role. Specifically, when harvesting takes place in the winter months the contamination risk is lower by a factor of 0.83 compared to the summer months. This can also be explained by the condition in which the cadaver is preserved, given the fact that the temperature, which is on average higher in summer than in winter, promotes a greater microbial growth, particularly if the donor died outside of hospital, as is the case for most of our donors.

In compliance with the existing literature (Deijkers et al. 1997; Ireland and Spelman 2005; Jashari et al. 2007; Paolin et al. 2017; Tabaku et al. 2004; Vehmeyer et al. 2002), *coagulase negative Staphylococcus* strains are most frequently isolated and the presence of these skin commensals is probably due to external contamination at the time of retrieval, i.e., leakage from the skin incisions which are made to access the thoracic and abdominal cavities, exposure to the environment during retrieval and handling. Bacterial strains from intestinal and upper airways flora were also found. These can be due to multiple causes, such as trauma consequences, or to the retrieval of CVT from the thoracic and abdominal cavities of donors who often present trauma-induced haemorrhagic effusions that can facilitate passive and active cross-contamination. It is clear that donor-related variables, such as gender, type, and period of the year of retrieval cannot be changed. Conversely, the results of our study can be an excellent starting point to improve tissue harvesting procedures.

In the light of the findings of the present study, we believe that to minimize the risk of bacterial contamination at retrieval, tissues should be procured with aseptic techniques, keeping the number of retrieval team members to a minimum. In addition, potential donors should be refrigerated as soon as possible, especially in summer, and cadaver time should be kept as short as possible.
